# Perioperative treatment with cilostazol reverses steatosis and improves liver regeneration after major hepatectomy in a steatotic rat model

**DOI:** 10.1038/s41598-025-87135-z

**Published:** 2025-01-22

**Authors:** Sebastian Holländer, Maximilian von Heesen, Gereon Gäbelein, Julie Mercier, Matthias W. Laschke, Michael D. Menger, Matthias Glanemann, Antonios E. Spiliotis

**Affiliations:** 1 Department of General Surgery, Vascular-, Visceral- and Pediatric Surgery, Saarland University Medical Center, 66421 Homburg, Germany; 2 Department of General- and Visceral Surgery, University Hospital Göttingen, 37075 Göttingen, Germany; 3 Institute for Clinical and Experimental Surgery, Saarland University, 66421 Homburg, Germany; 4 Department of Surgery, Campus Charité Mitte, Campus Virchow Klinikum, Charité Universitätsmedizin Berlin, 13353 Berlin, Germany

**Keywords:** Obesity, Non-alcoholic fatty liver disease, Hepatectomy, Cilostazol, Liver regeneration, Outcomes research, Preclinical research, Liver diseases

## Abstract

Cilostazol has previously been shown to reduce liver steatosis and enhance hepatic perfusion. We investigated the effects of cilostazol after major hepatectomy in a steatotic rat model. Six weeks prior to surgery, Sprague–Dawley rats were fed with a high-fructose diet. The treatment group received daily 5 mg/kg cilostazol. Seven days following the cilostazol treatment, all animals underwent 70% liver resection (PHX). Analysis of hepatic blood flow and microcirculation and immunohistochemical examinations were conducted 30 min after PHX (postoperative day [POD] 0) as well as on POD 1, POD 3 and POD 7. The weight of cilostazol-treated animals was significantly reduced compared to untreated controls after completion of the 6-week high-FRC diet. Furthermore, 41% macrovesicular steatosis was found in the control group compared to 8% in the cilostazol group. Hepatic arterial and portal venous perfusion were increased in the cilostazol group on POD 7. Lower liver enzyme release was found postoperatively in cilostazol-treated animals. Moreover, apoptosis and neutrophil infiltration were reduced after cilostazol treatment. Proliferation of hepatocytes and liver regeneration after PHX were significantly increased in the cilostazol group. Consequently, cilostazol should be evaluated as a novel strategy to reduce the rate of liver failure after PHX in steatotic liver.

## Introduction

Metabolic dysfunction-associated steatotic liver disease (MASLD) is nowadays the most prevalent chronic liver disease and a progressively growing epidemic worldwide^1,2^. The generic term MASLD summarizes several conditions and a sequence from simple steatosis to the aggressive necro-inflammatory form (non-alcoholic steatohepatitis, NASH) with a further transition to liver fibrosis and cirrhosis. The high prevalence of MASLD correlates with that of obesity, type II diabetes and insulin resistance, leading to deposition and liver accumulation of triglycerides and free fatty acids^3^. The calculated prevalence of MASLD varies across studies due to the use of different diagnostic methods and demographic factors and is estimated at 20–40% in the general population^1,4^ and up to 70–95%^5–7^ in obese individuals.

MASLD/NASH-related cirrhosis constitutes the second most common cause for liver transplantation in the United States and the fastest growing indication for liver transplantation in the last 20 years^8^. Furthermore, steatosis is considered a predominant cause of hepatocellular cancer in the United States and in Europe^8^. Consequently, liver surgery in patients with MASLD is a common surgical procedure with increased frequency during the last decades. However, although liver surgery generally presents excellent results, steatosis and obesity are associated with an increased risk for postoperative complications and mortality^9–11^.

Several pharmacological agents have been examined as perioperative treatments for liver regeneration following major hepatectomy. Among them, cilostazol, a phosphodiesterase-3 (PDE-3) inhibitor, has been associated with reduced liver steatosis and inflammation in a steatotic rat model^12^. Moreover, we previously investigated the effects of cilostazol in a non-steatotic major hepatectomy model and found that cilostazol increases hepatic blood perfusion and liver regeneration^13^. Furthermore, we showed that cilostazol reduces hepatocellular disintegration and inflammation after ischemia–reperfusion injury^14^. Based on these promising findings, we investigated in the present study the effects of cilostazol on hepatocellular damage, liver regeneration and inflammation after major hepatectomy in rats with steatosis induced by a high-fructose (high-FRC) diet.

## Materials and methods

Sprague–Dawley rats of both genders (body weight: 253 ± 4 g without significant differences between the groups, n = 28 female, n = 36 male; Institute for Clinical and Experimental Surgery, Saarland University, Homburg, Germany) were used for the experiments. The animals were housed at a constant temperature of 22 ℃ on a 12-h/12-h light–dark cycle and had free access to standard pellet food (Altromin, Lage, Germany) and tap water. The animals were divided into a cilostazol group (n = 32, 17 male) and a control group (n = 32, 19 male).

The study was performed in accordance with the European legislation on the protection of animals (Directive 2010/63/EU) and the National Institutes of Health guidelines for the Care and Use of Laboratory Animals^15^. All experiments and the experimental protocol were authorized by the local governmental animal protection committee (Landesamt für Verbraucherschutz, Saarbrücken, Germany; permission number 15/2015). Authors complied with the ARRIVE guidelines^16^.

### Experimental protocol

Six weeks prior to the surgical procedure, all animals were fed a high-fructose (FRC) diet (66% of total calories as fructose, 11% as fat and 19% as protein) obtained from Ssniff-Spezialdiäten GmbH (Soest, Germany). Seven days prior to surgery, animals (n = 32, cilostazol group) received 5 mg/kg cilostazol ((6-[4-(1-cyclohexyl-1H-tetrazol-5-yl) butoxy]-3,4-dihydro-2-(1H))-quinolinone; Schwarz Pharma, Monheim, Germany) via the drinking water. This treatment was continued throughout the postoperative period. The animals in the control group received water without cilostazol.

After the pretreatment period, all animals underwent a 70% liver resection (partial hepatectomy (PHX)). Hepatic blood flow and hepatic microvascular perfusion were analyzed at 30 min (postoperative day (POD) 0), 24 h (POD 1), 72 h (POD 3) and 168 h (POD 7) after PHX (n = 8 for each group and time point). After these in vivo measurements, a 2 mL blood sample was taken from the inferior vena cava and the animals were sacrificed during the anesthesia (140 mg/kg pentobarbital sodium as injection in the inferior vena cava). The remnant liver tissue was harvested and stored for further analyses.

### Surgical procedure

Prior to PHX, anesthesia was induced using 5% isoflurane, which was introduced via a gas line into a plastic box. Thereafter, the animals were placed in supine position on a heating pad with their body temperature maintained at 37 ℃. The anesthesia was conducted using 1–2% isoflurane and a sealing, funnel-shaped mask. For analgesia, tramadol (10 mg/kg body weight, Grünenthal GmbH, Aachen, Deutschland) was administered intraperitoneally.

After midline laparotomy, a 70% liver resection according to the technique described by Higgins and Anderson was performed^17^. After liver resection, the abdomen was closed with a double-layer running suture (Vicryl 4–0, Ethicon/Johnson & Johnson Medical Ltd, Livingston, UK). To measure hepatic blood flow and hepatic microvascular perfusion, animals were re-anesthetized using isoflurane and tramadol in the same way as described above. After relaparotomy, the hepatic artery and the portal vein were gently separated from the hepatoduodenal ligament.

### Ultrasonic flow measurements

An ultrasonic perivascular flow probe (0.5 V; Transonic Systems, Ithaca, New York, USA) was placed around the hepatic artery. Another flow probe (1.5R; Transonic Systems, Ithaca, New York, USA) was positioned around the portal vein. Hepatic arterial and portal venous blood flow were assessed simultaneously by use of a flow meter (T206 Animal Research Flowmeter; Transonic Systems, Ithaca, New York, USA). The detected blood flow was recorded for 10 min at each observation time point.

### Laser doppler flowmetry

After transection of the ligaments of the liver to minimize movements due to breathing excursions, microvascular perfusion of the liver parenchyma was evaluated with laser Doppler fluxmetry (PeriFlux System 500; Perimed, Inc., Hertfordshire, UK). For microvascular blood flow measurements, the fiberoptic probe of the laser Doppler flowmeter was placed on the surface of the right lateral liver lobe and fixed by means of a holder.

### Liver regeneration and body weight

Prior to the surgical procedure, the body weight of the animals was determined. The remnant liver tissue was harvested at 30 min, 24, 72 and 168 h after 70% hepatectomy, and the wet tissue weight was determined. An analysis of sham animals revealed a total liver weight of 3% of the body weight^18–20^. After hepatectomy, the relative liver weight, indicating the regeneration rate at the observation time points, was expressed as percentage and was calculated according to the following formula^13,21^:

Regeneration rate = [Remnant liver weight at given time point—(Estimated total liver weight at operation—Excised liver weight) / Total liver weight] × 100.

### Histology

At the end of the in vivo experiments, tissue samples were harvested from remnant liver lobes, fixed in 4% formalin for 2 days at 4°℃, and embedded in paraffin. Dehydrated, paraffin-embedded 3-µm sections were stained with hematoxylin–eosin and analyzed under light microscopy (Olympus BX60, Olympus Optical, Company Ltd., Japan). The light microscopic images were transferred to a desktop PC using a digital camera (Zeiss Axio Cam, Zeiss, Jena, Germany). Cytoplasmic vacuolization of hepatocytes, indicating hepatocellular steatosis, was scored in 20 high-power fields (HPF) by a semiquantitative score from 0 to 4 as described previously^14^: (0) none, (1) minimal (< 10% hepatocytes), (2) mild (10%–25% hepatocytes), (3) moderate (> 25%–50% hepatocytes) and (4) severe (> 50% hepatocytes).

The fat vacuolization area and mean vacuole diameter were calculated using the software ImageJ. The calculation of percentage area of fat vacuoles was performed the following way: First, the raw file was converted to 32-bit grayscale. Next, the white areas representing fat vacuoles and liver sinusoids were identified by applying the ‘threshold’ command in ImageJ. Subsequently, the ‘analyze particles’ command was employed to calculate the proportion of white areas as a percentage. It was assumed that the fat vacuoles are nearly perfect circles, and liver sinusoids were excluded from the calculation using the ‘circularity’ command.

To calculate the mean vacuole diameter, horizontal lines were drawn at 10 different representative points of the sample image. The program then measured the diameters of the vacuoles crossed by the lines and calculated the mean values for the respective test animals.

### Immunohistochemistry

To study cell proliferation and apoptotic cell death, proliferating cell nuclear antigen (PCNA) and cleaved caspase-3 were stained with indirect immunoperoxidase techniques^22,23^. For this purpose, deparaffinized sections were incubated with 3% H_2_O_2_ and 3% goat normal serum to block endogenous peroxidases and unspecific binding sites. A monoclonal mouse anti PCNA antibody (1:50; DakoCytomation, Glostrup, Denmark) and a polyclonal rabbit anti-rat cleaved caspase-3 antibody (Asp175, 1:100; Cell Signaling Technology, Frankfurt, Germany) were used as primary antibodies. The cleaved caspase-3 antibody detected endogenous levels of the short fragment (17/19 kD) of activated caspase-3 but not full-length caspase-3. Peroxidase-labeled goat anti-mouse and biotinylated goat anti-rabbit IgG antibodies were used as secondary antibodies. For streptavidin–biotin complex peroxidase staining (1:200, Labeled Streptavidin–Biotin 2 System horseradish peroxidase; DakoCytomation, Glostrup, Denmark) was used. 3,30-Diaminobenzidine (DakoCytomation, Glostrup, Denmark) was used as chromogen. Sections were counterstained with hemalaun and examined by light microscopy.

For the immunohistochemical detection of myeloperoxidase (MPO)-positive neutrophilic granulocytes, staining and incubation were carried out in the same way as for cleaved caspase-3 immunohistochemistry. A polyclonal rabbit anti-MPO antibody (1:100; Abcam, Cambridge, UK) was used as primary antibody. A biotinylated goat anti-rabbit IgG antibody (ready-to-use; Abcam, Cambridge, UK) served as secondary antibody. The biotinylated antibody was detected by peroxidase- labeled streptavidin (ready to use; Abcam, Cambridge, UK). 3-Amino-9-ethylcarbazole (Abcam, Cambridge, UK) was used as chromogen.

Sections were analyzed by counting caspase-3-, PCNA- and MPO-positive cells per HPF and given as the mean of 20 consecutive HPF. The immunohistological analyses were performed in randomly selected HPF of non-steatotic tissue.

### Blood analysis

After collection, the blood was centrifuged in an Eppendorf tube so that the serum could be skimmed off. The enzyme activity of alanine aminotransferase (ALT), aspartate aminotransferase (AST) and alkaline phosphatase (ALP) was then determined by spectrophotometry.

### Statistics

The collected data were recorded in Excel spreadsheets (Microsoft Office Excel 2007 Inc., Microsoft Corporation, USA). Statistical analysis was performed using SPSS (SPSS Version 23.0, IBM, Armonk, New York, USA). All data are presented as mean with standard error of the mean (SEM). After analysis of the normal distribution of data and homogeneity of variance, comparisons between 2 groups were made using a Student’s t-test (parametric data) or Mann–Whitney-U-test (non-parametric data). Differences were considered statistically significant for a value of p < 0.05.

## Results

### Body weight

The therapy with cilostazol did not affect the normal diet consumption of the rats and was not associated with a systemic toxicity. After completion of the high-FRC diet and pretreatment with cilostazol, the animals were weighed. A different body weight between cilostazol-treated animals and controls was detected. Specifically, cilostazol-treated animals were significantly lighter on average than untreated controls (cilostazol: 231.7 ± 3.5 g vs. control: 257.6 ± 5.6 g).

### Liver morphology

**Fig. 1:**
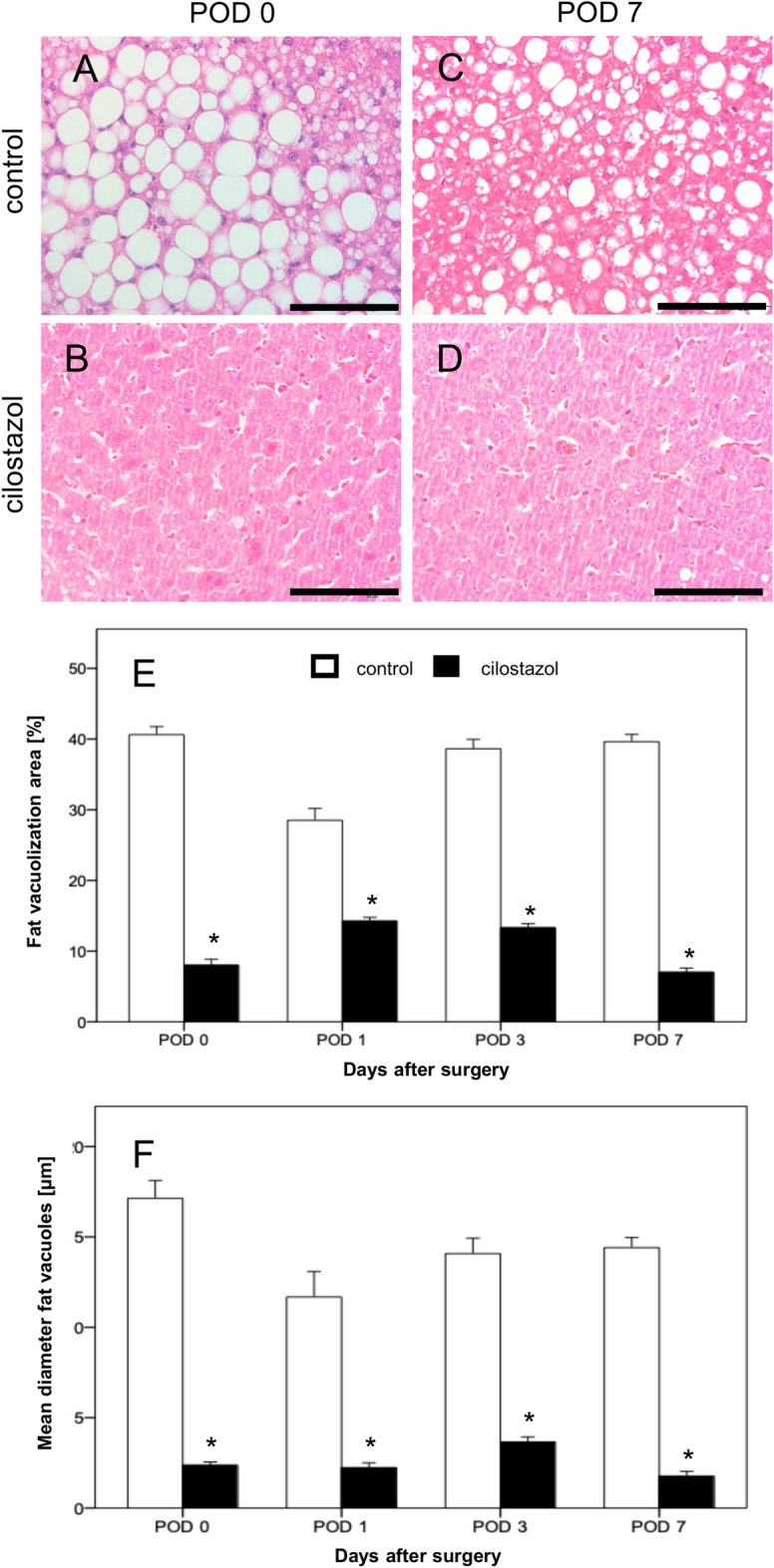
(**A**–**D**) Hematoxylin–eosin-stained liver sections of the control group (**A**, **C**) and the cilostazol group (**B**, **D**) on POD 0 and POD 7. Scale bars: 100 μm. (**E**, **F**): Fat vacuolization area (%) (**E**) and mean diameter fat vacuoles (µm) of livers in control (white bars, n = 32) and cilostazol-treated animals (black bars, n = 32). Mean ± SEM; *p < 0.05 vs. control.

A 6-week diet with high-FRC led to 8% macrovesicular steatosis in the cilostazol group compared to 41% in the control group on POD 0 (Fig. 1). Treatment with cilostazol significantly reduced the macrovesicular steatosis on all investigated days compared to the animals in the control group (Fig. 1).

### Hepatic blood flow

The measured values for arterial and portal venous blood flow as well as for hepatic microvascular perfusion presented large heterogeneity. The mean arterial blood flow on POD 3 in cilostazol-treated animals was significantly reduced compared to controls (Table 1). Although all other means did not differ significantly between the two groups, a trend towards better perfusion was seen on POD 7 in the cilostazol group. Specifically, the hepatic arterial and portal vein perfusion were documented increased by 60 and 37% in the cilostazol group compared to the control group, respectively.

**Table 1:** Hepatic arterial flow, portal venous flow, and microvascular perfusion in the control and cilostazol group on POD 0, POD 1, POD 3, and POD 7.

	Hepatic artery [µL/min/g BW]	Portal vein [µL/min/g BW]	Microvascular perfusion [aU]
POD 0			
Control	0.8 ± 0.2	26.8 ± 4.2	437.4 ± 55.3
Cilostazol	1.2 ± 0.3	41.0 ± 5.1	368.3 ± 20.8
POD 1			
Control	0.7 ± 0.1	56.6 ± 3.1	508.6 ± 25.2
Cilostazol	0.8 ± 0.1	37.3 ± 5.7	319.5 ± 36.2
POD 3			
Control	1.1 ± 0.1	42.8 ± 5.0	344.4 ± 32.7
Cilostazol	0.8 ± 0.1*	41.9 ± 3.6	416.7 ± 34.1
POD 7			
Control	0.6 ± 0.1	27.5 ± 3.4	365.1 ± 25.9
Cilostazol	1.5 ± 0.1	43.1 ± 4.9	357.3 ± 50.4

Mean ± SEM, * p < 0.05 vs. control.

### Liver enzyme release after PHX

**Fig. 2:**
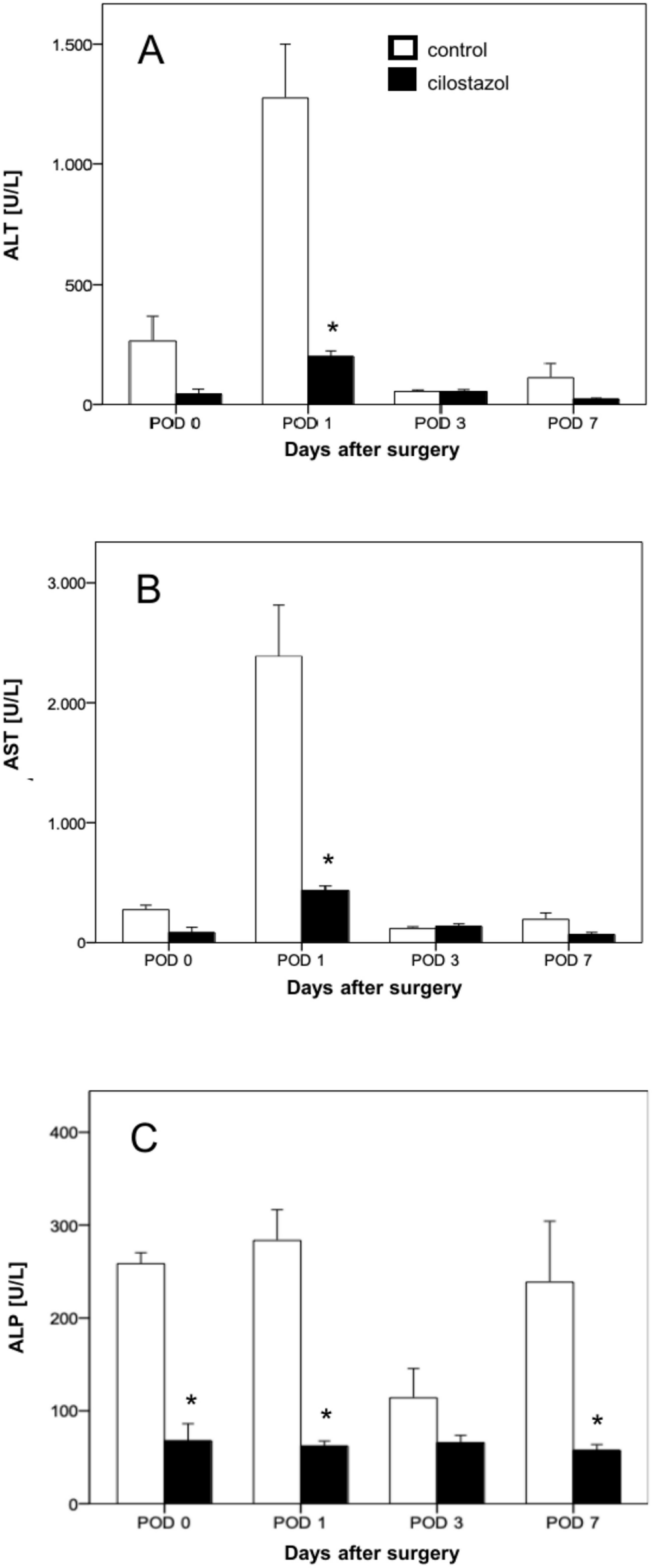
(**A**–**C**) Serum concentration (U/l) of ALT (**A**), AST (**B**) and ALP (**C**) on POD 0, POD 1, POD 3 and POD 7 in control (white bars, n = 32) and cilostazol-treated animals (black bars, n = 32). Mean ± SEM, *p < 0.05 vs. control.

On POD 0, immediately after 70% hepatectomy, there was a noticeably increased release of the hepatocellular enzymes ALT, AST and ALP in the blood serum. The enzyme release of ALT and AST increased to a maximum on POD 1 and decreased again on POD 3 and POD 7. A lower enzyme release was found on POD 0, POD 1 and POD 7 in animals treated with cilostazol. The difference on POD 1 was statistically significant (Fig. 2). An increased release of ALP was detected immediately after PHX. The enzyme concentration remained elevated throughout the experiments. In animals treated with cilostazol significantly lower concentrations of ALP were found at POD 0, POD 1 and POD 7 (Fig. 2).

### Inflammatory response

**Fig. 3:**
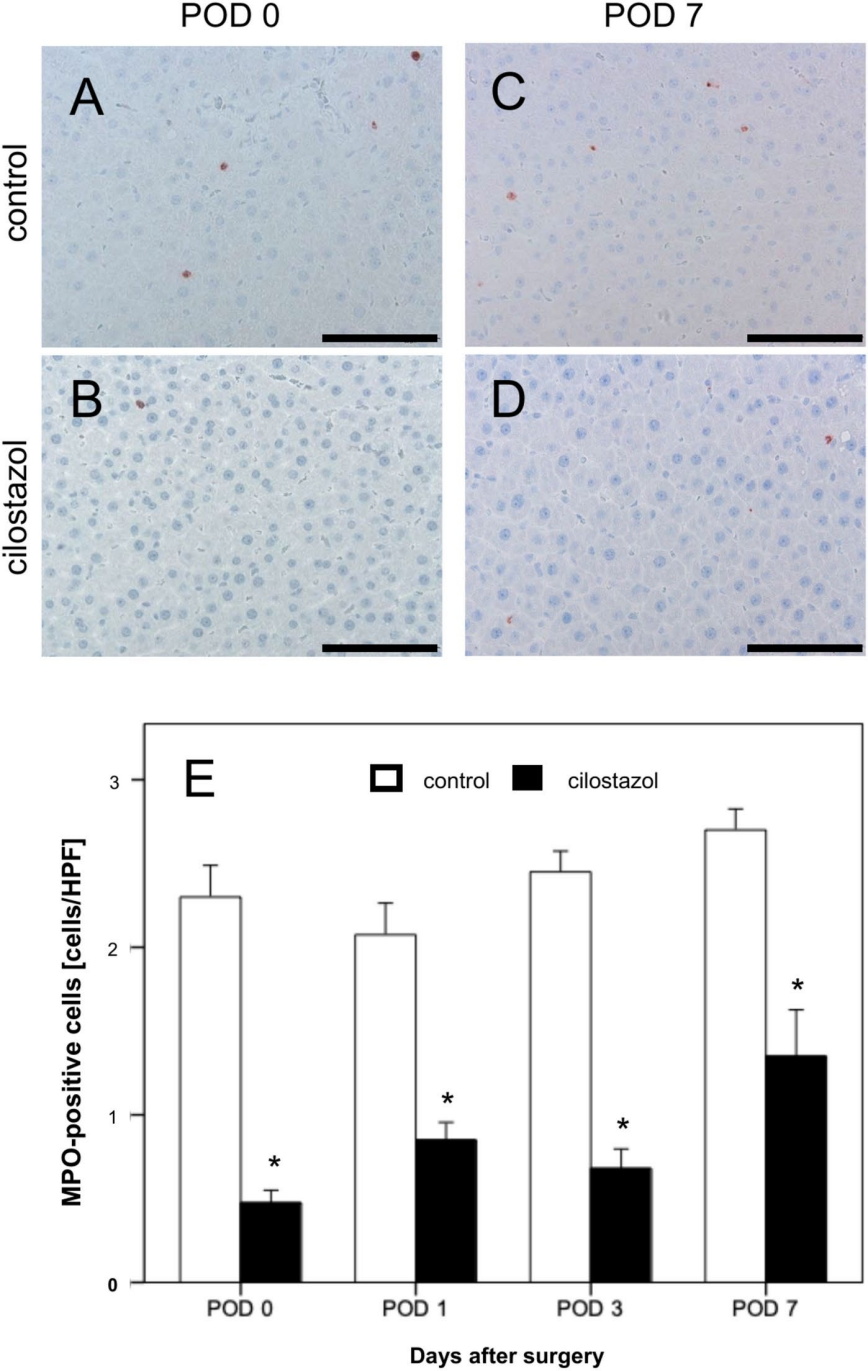
(**A**–**D**) MPO-stained liver sections of the control group (**A**, **C**) and the cilostazol group (**B**, **D**) on POD 0 and POD 7. Scale bars: 100 μm. (**E**) MPO-positive cells per HPF in residual liver tissue on POD 0, POD 1, POD 3 and POD 7 in control (white bars, n = 32) and cilostazol-treated animals (black bars, n = 32). Mean ± SEM, * p < 0.05 vs. control.

In the control group, two to three MPO-positive neutrophilic granulocytes per HPF were detected on POD 0–7. A significant reduction in the inflammatory response was found in the cilostazol group on all examination days (Fig. 3).

### Apoptotic cell death

**Fig. 4:**
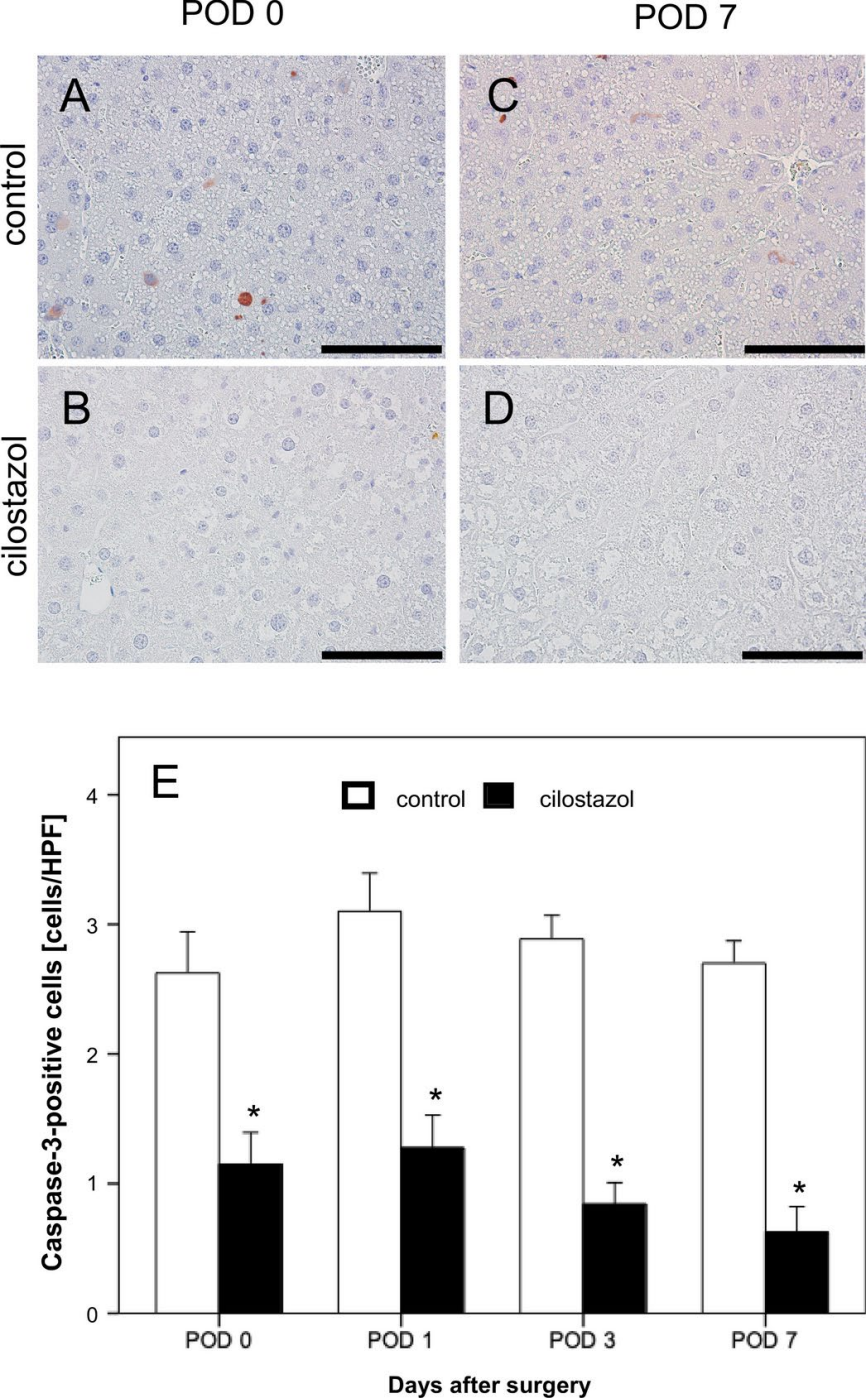
(**A**–**D**) Caspase-3-stained liver sections of the control group (**A**, **C**) and the cilostazol group (**B**, **D**) on POD 0 and POD 7. Scale bars: 100 µm. (**E**) Caspase-3-positive cells per HPF in residual liver tissue on POD 0, POD 1, POD 3 and POD 7 in control (white bars, n = 32) and cilostazol-treated animanls (black bars, n = 32). Mean ± SEM, * p < 0.05 vs. control.

As an additional marker for hepatic injury, cleaved caspase-3-positive apoptotic hepatocytes were assessed. Overall, a low number of apoptotic hepatocytes was found. Although only two to three cells out of 100 hepatocytes were apoptotic in the control animals, this rate was significantly increased compared to the cilostazol group (Fig. 4).

### Liver regeneration

**Fig. 5:**
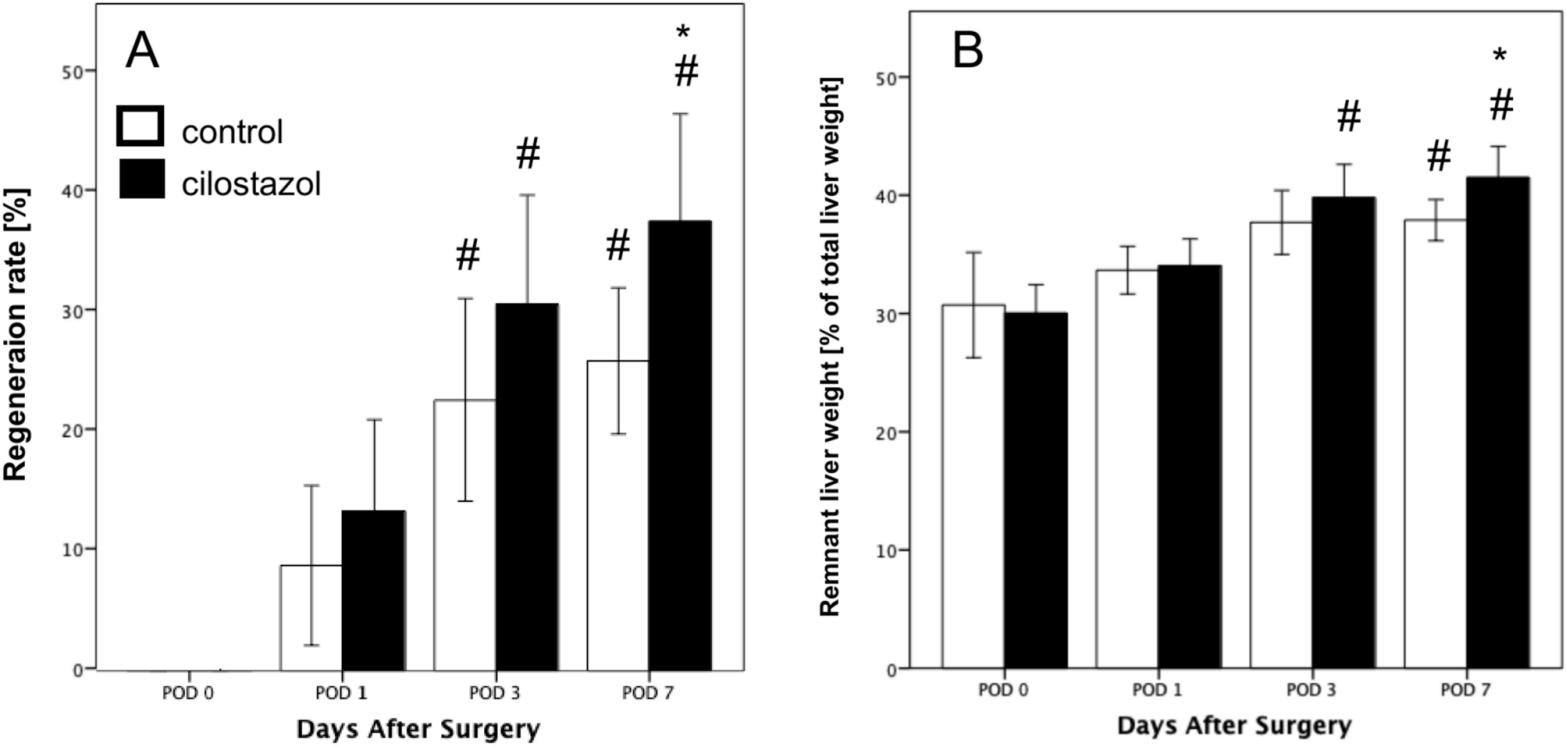
(**A**, **B**) Regeneration rate (**A**) and remnant liver weight as percentage of the total liver weight (**B**) on POD 0, POD 1, POD 3, and POD 7 in control (white bars, n = 32) and cilostazol-treated animals (black bars, n = 32). Mean ± SEM, ^#^ p < 0.05 vs. POD 0, * p < 0.05 vs. control.

Both groups exhibited consistent liver tissue regeneration throughout the observation period. While the regeneration rate was comparable between the two groups during the first three observation time points, a statistically significant increase in regeneration was observed on POD 7 in cilostazol-treated animals compared to controls (Fig. 5A). In both groups, remnant liver weight was statistically increased on POD 7 when compared to the weight on POD 0 (Fig. 5B).

### Proliferation

**Fig. 6:**
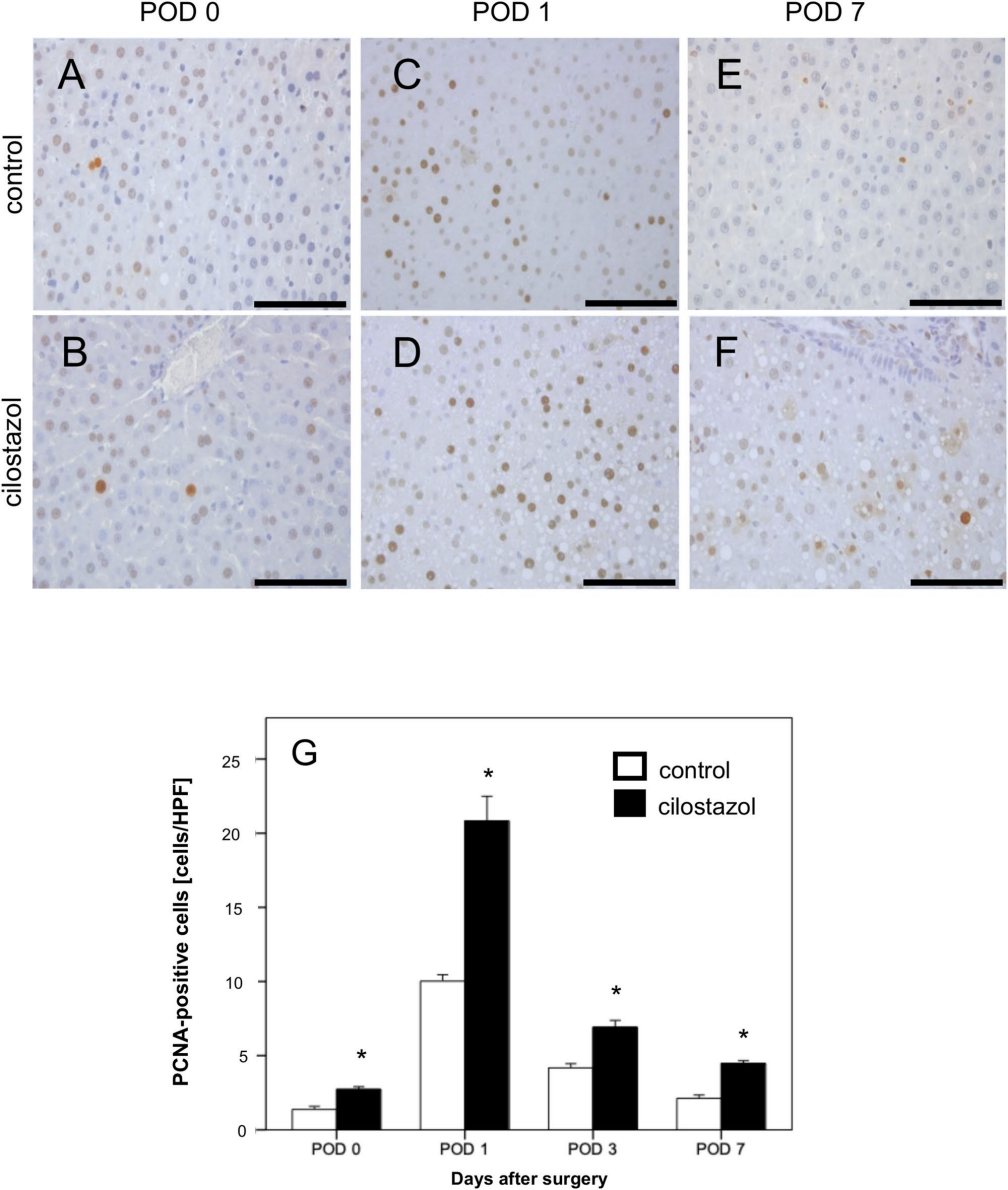
(**A**–**F**) PCNA-stained liver sections of the control group (**A**, **C**, **E**) and in the cilostazol group (**B**, **D**, **F**) on POD 0, POD 1 and POD 7. Scale bars: 100 µm. (**G**) PCNA-positive cells per HPF in residual liver tissue of control (white bars, n = 32) and cilostazol-treated animals (black bars, n = 32). Mean ± SEM, * p < 0.05 vs. control.

The rate of PCNA-positive cells as a marker for cell division of hepatocytes reached its maximum on POD 1 and decreased again on the following days (POD 3 and POD 7). Treatment with cilostazol led to a significant increase of PCNA-positive cells when compared to controls (Fig. 6). This effect also reached its maximum on POD 1. On average, there were approximately twice as many PCNA-positive cells in the cilostazol group as in the control group. Interestingly, the proliferation rate was already significantly increased in the cilostazol group on POD 0 compared to the control group.

## Discussion

The per capita consumption of fructose has increased significantly worldwide since the late 1960s and early 1970s, mainly due to the development of sweet corn-based syrups, known as High Fructose Corn Syrup (HFCS). Between 1970 and 2000, consumption of HFCS increased more than 110-fold, with countries like the United States seeing an increase in consumption from 0.292 kg/person/year to 33.4 kg/person/year. This increase in fructose consumption far exceeds changes in intake of any other food group and is considered as a major contributor to the rise in MASLD^24^ and metabolic syndrome in general.

MASLD and NASH are associated with a predisposition to carcinogenesis and the development of hepatocellular cancer, while also constituting one of the most common etiologies for liver transplantation^8,25^. In addition, MASLD/NASH is considered a risk factor for postoperative complications in patients undergoing major hepatectomy or transplantation^8,25^. The unfavorable postoperative course of these patients is attributed to the fact that steatosis enhances the risk of hepatic reperfusion injury following major hepatectomy and impedes liver regeneration and functional recovery^10,26–28^.

In an experimental study has been documented the impaired liver regeneration following 70% PHX in a high-FRC steatotic model^29^. Based on these results and the reported association between high-FRC diet and impaired liver regeneration, we herein investigated the effects of cilostazol on hepatocellular damage, liver regeneration and inflammation after major hepatectomy in a high-FRC-induced steatotic model.

At first, we documented a significant decrease in body weight of the treated animals 6 weeks following the high-FRC diet compared to the control group. This result is attributed to the known mechanism of cilostazol-induced improvement in glucose metabolism, lipid metabolism, and thermogenesis process of white adipose tissue, leading to weight loss^30,31^.

We found that perioperative cilostazol treatment improves the postoperative outcomes after PHX. Cilostazol-treated rats exhibited less macrovesicular steatosis at the time of PHX when compared to controls. Furthermore, cilostazol treatment limited the inflammatory response, reduced liver cell damage, improved liver regeneration and reduced apoptotic cell death after PHX. Hence, our results demonstrate a beneficial effect of cilostazol on steatotic liver, which is more sensitive to surgical trauma. In our opinion, the significant reduction of steatosis is the main reason for the observed effects and provides evidence for an effective perioperative treatment.

The mechanism of action of cilostazol is based on inhibition of PDE-3 and subsequent activation of PKA pathways in hepatocytes. Biochemical key mechanisms of fat accumulation, oxidative stress and consecutive inflammation after a high-FRC diet are counteracted by cilostazol treatment. Specifically, cilostazol, as a type III PDE-3 inhibitor with partial type V phosphodiesterase-5 activity, increases the intracellular 3′-5′-cyclic adenosine monophosphate (cAMP) level by blocking its hydrolysis^32^. Elevated cAMP levels and the cellular mechanisms triggered by PKA pathways appear to be an effective counterbalance to the mechanisms of hepatocellular fatty degeneration.

The beneficial effect of cilostazol on steatotic livers was demonstrated in our study, where cilostazol-treated rats showed a significantly increased regeneration rate on POD 7. Immunohistochemical examination confirmed enhanced hepatocyte proliferation, as PCNA-positive cells were significantly increased in the cilostazol group at all observation times. Consistent with these findings, we previously demonstrated that cilostazol treatment is associated with an increased liver regeneration rate of approximately 30 and 35% on POD 3 and 6, respectively, after 70% hepatectomy in non-steatotic rat livers^13^.

Portal hyperperfusion has been identified as a critical factor leading to hepatic failure in patients undergoing extended hepatectomy or small-for-size transplantation^33^. Excessive portal perfusion can reduce hepatic arterial flow and oxygen supply^13^. Portal hyperperfusion decreases the concentration of interstitial adenosine, which reduces its vasodilatory effect on the hepatic artery. We previously demonstrated that cilostazol enhances portal venous flow, hepatic arterial perfusion and parenchymal microcirculation^13^. Specifically, cilostazol-treated animals maintained adequate hepatic arterial perfusion despite elevated portal venous flow after liver resection^13^. In the present study, hepatic arterial perfusion was increased by 60% in the cilostazol group on POD 7 compared to the control group, while portal vein perfusion was increased by 37%. Although these results were not statistically significant, a trend of improved blood flow in the cilostazol group after major hepatectomy was recorded. However, we did not observe a cilostazol-induced enhancement of the hepatic microcirculation in our steatotic model.

According to our previous results, cilostazol treatment is associated with two mechanisms of action that lead to increased portal venous and hepatic arterial perfusion. The first mechanism is the stimulation of angiogenesis-related genes and endothelial nitric oxide synthase mRNA^13^. The second mechanism is the inhibition of adenosine uptake, which enhances its interstitial concentration, leading to a maintenance of the vasodilatory capacity and an enhancement of the hepatic arterial flow^13^.

Our results also showed that cilostazol treatment significantly prevents apoptotic cell death of hepatocytes. Apoptosis is a main mechanism of cellular death in hepatic failure and reperfusion injury^34^. Following reperfusion injury, high apoptotic activity is expected in the early postoperative phase, which remains slightly increased after 24 hours^35^. This pattern of apoptotic activity was detected in the cilostazol group, where the apoptotic ratio reached the highest level on POD 1 and the lowest level on POD 7. At all observation points, apoptosis was significantly increased in the control group compared to the treatment group, indicating the hepatoprotective effect of cilostazol.

In addition, we observed a significant reduction of MPO-positive cells in the treatment group compared to the control group. The granulocytic inflammatory activity in the remaining liver tissue after PHX was statistically decreased in the cilostazol group. The upregulation of PKA inhibits the extracellular signal-regulated kinases-1/2 and P38 mitogen-activated protein kinases, which suppresses the release of proinflammatory cytokines, such as tumor necrosis factor alpha by Kupffer cells^36^. Via the same signaling pathways, the upregulation of cAMP and PKA probably also leads to an increased release of anti-inflammatory interleukin (IL)-10 and to a reduction in the production of pro-inflammatory cytokines, such as IL-1 and MPO^37^.

Several pharmacological approaches have been examined for their hepatoprotective effects after major hepatectomy in rodent models. One such approach is the use of the PDE-3 inhibitors amrinone and olprinone, which have shown beneficial outcomes in liver regeneration after hepatectomy^38,39^. In swine hepatectomy models, preoperative treatment with olprinone attenuated the elevation of portal vein pressure, reduced ischemia–reperfusion injury and enhanced hepatic regeneration^33^. Similarly, in a 90% hepatectomy model, olprinone attenuated the increase in portal venous pressure, which was attributed to up-regulation of endothelial nitric oxide synthase. Furthermore, olprinone exerted hepatoprotective effects on endothelial cell injury and apoptosis^39^. In the literature, several other treatment strategies have been described, which could have a beneficial effect on the steatotic liver. Omega-3 polyunsaturated fatty acids (Ω3FA) have also been utilized to suppress steatosis. In a rat obesity model, Ω3FA showed increased regeneration of liver weight one week following a 70% hepatectomy. The authors examined the reperfusion injury after a major hepatectomy and concluded that the anti-inflammatory properties of Ω3FA protect from hepatic injury^26^. Epidermal growth factor receptor (EGFR) downregulation has also been reported as a crucial factor of delayed hepatic regeneration in MASLD^40^. Accordingly, EGFR replacement treatment improved the regenerative response of the fatty liver in an obesity mouse model after 70 and 80% hepatectomy. However, further studies are required to compare the different hepatoprotective agents and to identify the best perioperative treatment.

The majority of liver resections in humans are performed due to tumors. This raises the question of whether cilostazol, while potentially improving surgical outcomes, may also promote tumor growth. However, Strowitzki et al. showed that cilostazol treatment is not associated with tumor progression during liver regeneration after major hepatectomy. Furthermore, the proliferation rate of tumor cells was not affected, whereas reduced tumor vascularization was found in rats treated with cilostazol^41^.

The present study has several limitations. Following the 3R principle (replacement, reduction and refinement), the experiments were performed only in a limited number of animals, which may affect the results and preclude more statistical analysis of the obtained data. Specifically, the absence of a sham group can lead to a misinterpretation of the results on neutrophil infiltration, apoptosis, or proliferation in the setting of a diet-induced steatohepatitis. Moreover, the results are limited to the steatotic liver, and further studies are highly desirable to elucidate the underlying biochemical pathways, the effectiveness and role of cilostazol in tumor models with steatosis as well as prior to transplantation. Finally, the regeneration rate was compared in two groups with significant different steatotic profile at the time of surgery as provided in histological examinations on POD 0. Consequently, this different pattern of steatosis could also be a causal factor of decreased regeneration in the control group.

## Conclusion

Perioperative treatment with cilostazol represents an effective pharmacological strategy in major hepatectomy, as it significantly reduces neutrophil infiltration and hepatocellular damage, while also improving liver regeneration through a reduction of pre-existing steatosis.

## Data Availability

The data that support the findings of this study are available from the Institute for Clinical and Experimental Surgery in Saarland University (https://www.uniklinikum-saarland.de/de/einrichtungen/kliniken_institute/chirurgie/experimentalchirurgie). Further information is available from the corresponding author upon request.
